# In vitro assessment of the antimicrobial efficacy of chitosan nanoparticles against major fish pathogens and their cytotoxicity to fish cell lines

**DOI:** 10.1111/jfd.13212

**Published:** 2020-07-06

**Authors:** Fatma Ahmed, Faiza M. Soliman, Mohamed A. Adly, Hamdy A.M. Soliman, Mansour El‐Matbouli, Mona Saleh

**Affiliations:** ^1^ Clinical Division of Fish Medicine University of Veterinary Medicine Vienna Austria; ^2^ Department of Zoology Faculty of Science Sohag University Sohag Egypt

**Keywords:** antimicrobial efficacy, chitosan nanoparticles, cytotoxicity, disrupting interaction, fish cell lines, fish pathogens

## Abstract

Nanotechnology is an emerging avenue employed in disease prevention and treatment. This study evaluated the antimicrobial efficacy of chitosan nanoparticles (CSNPs) against major bacterial and oomycete fish pathogens in comparison with chitosan suspension. Initially, the minimum inhibitory concentrations (MIC, MIC_90_) were determined and the per cent inhibition of bacterial growth was calculated. Subsequently, the minimum bactericidal concentrations (MBCs) were determined. The time‐dependent disruptions of CSNP‐treated pathogens were observed via transmission electron microscopy (TEM), and the effect of CSNPs on the viability of two fish cell lines was assessed. No antimicrobial effect was observed with chitosan, while CSNPs (105 nm) exhibited a dose‐dependent and species‐specific antimicrobial properties. They were bactericidal against seven bacterial isolates recording MBC values from 1 to 7 mg/ml, bacteriostatic against four further isolates recording MIC values from 0.125 to 5 mg/ml and fungistatic against oomycetes recording MIC_90_ values of 3 and 4 mg/ml. TEM micrographs showed the attachment of CSNPs to the pathogenic cell membranes disrupting their integrity. No significant cytotoxicity was observed using 1 mg/ml CSNPs, while low dose‐dependent cytotoxicity was elicited by the higher doses. Therefore, it is anticipated that CSNPs are able to compete and reduce using antibiotics in aquaculture.

## 1 INTRODUCTION

Fish pathogens causing disease outbreaks are considered a major threat alarming the aquaculture industry, especially after the emergence of several antibiotic‐resistant bacterial pathogens. The maintenance of the farmed fish healthy and free of diseases is economically and ecologically essential. However, the excessive use of antimicrobial drugs, including antibiotics, promotes the foundation of antibiotic‐resistant pathogens, which is a high threat to the aquaculture industry (Cabello et al., 2013; Pelgrift & Friedman, 2013). Nanotechnology is an emerging avenue that provides a modern and innovative approach for utilization of the nano‐sized particles in novel applications for fish disease diagnostics and therapy (Shaalan et al., 2018; Shaalan, Sellyei, El‐Matbouli, & Székely, 2020). Recently, the technology of nanomedicine has gained wide importance and was employed for several therapeutic applications (Cavalieri, Tortora, Stringaro, Colone, & Baldassarri, 2014; Shaalan, Saleh, El‐Mahdy, & El‐Matbouli, 2016). Nanogold, silver and zinc oxide showed antimicrobial activities combating several fish pathogens and enabling alternative controlling of disease outbreaks (Saleh, Kumar, Abdel‐Baki, Al‐Quraishy, & El‐Matbouli, 2016; Shaalan, El‐Mahdy, Theiner, El‐Matbouli, & Saleh, 2017). However, nanoparticles of biodegradable biopolymers that could be prepared by simple methods without organic solvents are preferable for the biological applications (Agarwal et al., 2018). These biodegradable nanoparticles are small solid colloidal particles having a size range of 10–1000 nm that provides large surface areas and unique physical and chemical properties allowing a wide range of applications (Du, Niu, Xu, Xu, & Fan, 2009). It is worth mentioning that nanoparticles of high antimicrobial ability are considered the most advanced and promising reagents reducing the usage of the antimicrobial drugs, including antibiotics in combating several pathogens (Shaalan et al., 2016).

In addition to being involved in some biological applications (Chung et al., 2004), chitosan and its nanocomposites were incorporated in the medical applications as well due to their antimicrobial effects against several microorganisms (Ma, Garrido‐maestu, & Jeong, 2017). Chitosan [poly‐(b‐1/4)‐2‐amino‐2‐deoxy‐D‐glucopyranose] is a natural biodegradable polysaccharide, composed of randomly distributed chains of b‐(1–4) D‐glucosamine and N‐acetyl‐D‐glucosamine, and can be prepared by the alkaline deacetylation of the chitin forming the crustacean shells (Du, Xu, Xu, & Fan, 2008). Owing to its poor solubility above pH 6.5, chitosan exhibits its antimicrobial effects only in acidic media and shows higher efficacy at lower pH values (Qi, Xu, Jiang, Hu, & Zou, 2004). Chitosan antimicrobial efficacy is influenced by its polycationicity in acidic media, in addition to some physicochemical properties including its molecular weight, degree of polymerization and solvents (Du et al., 2009; Qi et al., 2004). In vitro, chitosan and chitosan nanoparticles (CSNPs) have been reported for the bacterial growth inhibition (de Paz, Resin, Howard, Sutherland, & Wejse, 2011) and for the inhibition of fungal growth (Ing, Zin, Sarwar, & Katas, 2012; Tayel et al., 2010). In vivo, chitosan is well known by its biosafety and low toxicity for the aquaculture industry in comparison with other natural polysaccharides (Abdel‐Ghany & Salem, 2019). Therefore, chitosan and CSNPs had been reported in a wide range of biomedical applications in aquaculture (Ahmed et al., 2019). However, more investigations on the antimicrobial effect of CSNPs against fish pathogens are still required.

This study aimed at investigating the efficacy of CSNPs against major fish bacterial and oomycete pathogens via detecting their corresponding active antimicrobial doses. This gives a new horizon for the evaluation of CSNP ability to combat fish diseases and decrease their threat to the aquaculture industry.

## 2 MATERIALS AND METHODS

### 2.1 Preparation of CSNPs

Low molecular weight chitosan with 90% deacetylation degree, sodium tripolyphosphate (STTP), acetic acid (HOAc) and sodium hydroxide (NaOH) was purchased from Sigma‐Aldrich, Austria. CSNPs were prepared via the ionic gelation method according to Qi et al. (2004) with minor modification. Briefly, ionic solution (0.5%, W/V) of chitosan was prepared by dissolving the powder in dilute aqueous HOAc (1%, V/V) via continuous magnetic stirring until the clear, transparent appearance. Then, the pH was raised from (3.7–3.9) to (4.6–4.8) via titration with NaOH (10 N). An aqueous STPP solution (0.25%, W/V) was prepared in deionized distilled water. The milky colloidal nanoparticle suspension was formed spontaneously by 1:3 dropwise addition of STPP into chitosan solution, under continuous magnetic stirring at room temperature for 2 hr allowing more ionic cross‐linking interaction between the two oppositely charged ions. The formed CSNPs were collected via centrifugation at 4,000 × *g* for 30 min at room temperature, and the supernatants were discarded. The purification of the nanoparticles from any extra NaOH was performed by resuspending the precipitated CSNPs in distilled water, extensive rinsing and collecting again via centrifugation at 4,000 × *g* for 30 min at room temperature. The collected gel‐like CSNP colloids were freeze‐dried and stored at 4ºC before further use or analysis. The lyophilized CSNPs were ground and resuspended either in distilled water for characterization or in the pathogen‐specific broth media for the antimicrobial assays (Du et al., 2009; Du et al., 2008).

### 2.2 Characterization of the prepared CSNPs

#### 2.2.1 Particle size and size distribution

Particle size and size distribution were estimated based on the dynamic light scattering (DLS) by using Malvern Zetasizer Nano ZS® device. This characterization analysis was conducted in triplicate on diluted CSNP suspensions in deionized distilled water. The DLS was measured under 90º scattering angle at 25ºC (Du et al., 2009; Du et al., 2008; Qi et al., 2004).

#### 2.2.2 Microscopic assay

Surface morphology of the prepared CSNPs including particle shape and aggregates was examined by scanning electron microscopy (*SEM*) (Phillips 500, Germany). A small amount from the ground freeze‐dried CSNPs was suspended in distilled water and mounted on a metal *SEM* stub using double‐sided adhesive tape at 50 mA for 6 min. The mounted sample was coated with platinum in a sputter coater under vacuum (Azizi et al., 2010; Divya, Vijayan, George, & Jisha, 2017). The stub containing the sample was placed in the *SEM* chamber, and the photomicrograph was taken at an acceleration voltage of 15 kV.

Particle morphology, along with the average particle diameter of the prepared CSNPs, was estimated via transmission electron microscopy (TEM) (EM 900, Zeiss, Oberkochen, Germany). A diluted suspension of the freeze‐dried CSNPs was deposited on a copper grid coated with carbon and dried at room temperature. After dryness, the sample was stained by the phosphate tungsten acid‐negative stain and examined at 80 kV accelerating voltage. Image SP Viewer® software was used to measure the diameters of the particles in the sample (Mohammadpour et al., 2012).

### 2.3 Assessment of the antibacterial efficacy of CSNPs

#### 2.3.1 Bacterial strains and growth conditions

Aliquots of sixteen fish pathogenic bacterial isolates related to seven different genera (*Aeromonas*, *Pseudomonas*, *Edwardsiella*, *Yersinia*, *Francisella*, *Citrobacter* and *Vibrio*) were tested for their sensitivity to CSNPs. *Aeromonas salmonicida* subsp. *salmonicida* (DSMZ 19,634) was purchased from the German Collection of Microorganisms and Cell Cultures, Germany. *Edwardsiella ictaluri* (93–146) was obtained as a gift from Prof. Dr. Lawrence, CVM College of Veterinary Medicine, Mississippi State University, USA. Both of *Vibrio harveyi* and *Vibrio alginolyticus* isolates were provided by Dr. Reza Ghanei‐Motlagh, Department of Clinical Sciences, Faculty of Veterinary Medicine, Shahid Chamran University of Ahvaz, Ahvaz 61357–831351, Iran. The other bacterial strains were isolated in our laboratory from clinically infected fish and identified morphologically, biochemically and molecularly, and then kept in our Microbank in the Clinical Division of Fish Medicine, University of Veterinary Medicine, Vienna, Austria. Gram staining confirmed the Gram‐negative isolates, and then, they were identified individually via MALDI and subjected to confirmatory testing by API, which showed an overall correct identification. API 20E showed positive predictive values for *Edwardsiella*, *Yersinia* and *Citrobacter* isolates, API 20NE showed positive predictive values for *Aeromonas*, *Pseudomonas* and *Vibrio* isolates, while API ZYM showed positive predictive values for the *Francisella* isolate. Finally, the identity of all the bacterial isolates was confirmed molecularly by amplifying the gene coding for bacterial 16S rRNA using the 63f (5’‐CAGG CCTAACACATGCAAGTC‐3`) and 1387r (5’‐CGGCGGWGTGTACAAGGC‐3`) primers according to Marchesi et al. (1998). Amplified products were gel‐purified and subjected to sequencing. Sequence analysis by BLASTn revealed 97%–99% identity of each isolate sequence with its target bacterial species sequence. The scientific names and the resources of all the isolates used for our study are presented in Table 1.

**Table 1:** Antibacterial activity of CSNP suspensions: minimum inhibitory concentrations (MICs) and minimum bactericidal concentrations (MBCs) in mg/ml

Bacterial isolate	Origin	CSNPs (mg/ml)	
		MIC	MBC
*Pseudomonas fluorescens* (10/7/13)	Koi (*Cyprinus rubrofuscus*)	0.125	1
*Aeromonas hydrophila*	Common carp (*Cyprinus carpio)*	0.5	2
*Yersinia ruckeri* (BC 74)	Rainbow trout (*Oncorhynchus mykiss*)	1	3
*Pseudomonas putida* (18/225)	Arctic char (*Salvelinus alpinus*)	1	7
*Aeromonas caviae*	Rainbow trout (*Oncorhynchus mykiss*)	1	–
*Aeromonas veronii* (25.4.)	Rainbow trout (*Oncorhynchus mykiss*)	1	–
*Francisella noatunensis* subsp. *orientalis* (11. 2015)	Ornamental cichlid fish	2	–
*Aeromonas salmonicida* subsp. *salmonicida* (DSMZ (19,634)†	Salmon	3	6
*Edwardsiella tarda* (12/2014)	Discus (*Symphysodon sp*.)	3	4
*Edwardsiella ictaluri* (93–146) ^‡^	Channel catfish	3	‐
*Pseudomonas aeruginosa* (9596/11)	Wels catfish (*Silurus glanis*)	5	5
*Citrobacter freundii* (15597/11)	Wels catfish (*Silurus glanis*)	–	–
*Vibrio vulnificus* (19/035)	Green chromis (*Chromis viridis*)	–	–
*Vibrio parahaemolyticus* (09/13 135)	Red lionfish (*Pterois volitans*)	–	–
*Vibrio alginolyticus ^§^*	Asian sea bass (*Lates calcarifer*)	–	–
*Vibrio harveyi ^§^*	Asian sea bass(*Lates calcarifer*)	–	–

Note

(†): The isolate was purchased from the German Collection of Microorganisms and Cell Cultures. (‡): The isolate is a gift from Prof. Dr. Lawrence, CVM College of Veterinary Medicine, Mississippi State University, USA. (§): The isolate was provided by Dr. Reza Ghanei‐Motlagh, Department of Clinical Sciences, Faculty of Veterinary Medicine, Shahid Chamran University of Ahvaz, Ahvaz 61357–831351, Iran. (–): The value was not reached at all doses examined during the current study.

The bacterial strains were grown on Müller‐Hinton (MH) agar (Sigma‐Aldrich, Austria) plates except for *Francisella noatunensis* subsp. *orientalis*, which was grown on cystine heart agar (Sigma‐Aldrich, Austria) supplemented with 2% horse blood after being inoculated in cystine heart broth (Sigma‐Aldrich, Austria) (Shaalan et al., 2017). All agar plates were incubated in a shaker incubator at 144 revolutions per minute (rpm) at 22°C overnight, except for *F. noatunensis* subsp. *orientalis* which required five days of incubation. *A. salmonicida* was incubated at 15°C for 48 hr (Shaalan et al., 2017), and Vibrios were incubated at 37ºC for 18–24 hr (ArunKumar, LewisOscar, Thajuddin, & Nithya, 2019; Najiah, Lee, Hassan, Shariff, & Mohd‐Azmi, 2003).

#### 2.3.2 Bacterial growth inhibition assay

A preliminary test to investigate the bacterial growth inhibition ability of chitosan and the prepared CSNP suspensions against the selected bacterial isolates was conducted following (Shaalan et al., 2017) with minor modification. Under aseptic conditions, a single colony from each bacterial isolate was inoculated in Müller‐Hinton (MH) broth (Sigma‐Aldrich, Austria) except for *F. noatunensis* subsp. *orientalis*, which was inoculated in modified Müller‐Hinton II cation‐adjusted broth (Sigma‐Aldrich, Austria), enriched with 2% IsoVitaleX (Becton, Dickinson). The inoculated broth media were incubated in a shaking incubator (144 rpm) at the same conditions described above. The bacterial suspensions were adjusted to match 0.5 at OD_600_ on a proper spectrophotometer (Eppendorf BioPhotometer®, Eppendorf, Hamburg, Germany) and then diluted to reach a final working concentration of 10^7^ CFU/ml.

Under aseptic conditions, chitosan and CSNP powders were accurately weighted (Xs Balance mod. 224‐220 gr.‐0.1 mg) and suspended directly in sterile MH broth and modified Müller‐Hinton II cation‐adjusted broth, enriched with 2% IsoVitaleX to obtain stock suspensions of 1 mg/ml (Du et al., 2008; Qi et al., 2004). In separate falcon tubes, the bacterial suspensions were inoculated into the chitosan and CSNP suspensions to achieve final concentrations of 1 × 10^5^ CFU/mL (Du et al., 2009), and were incubated at the same conditions described above. Un‐inoculated autoclaved MH broth or modified Müller‐Hinton II cation‐adjusted broth, enriched with 2% IsoVitaleX, served as blank controls. Inoculated MH broth or modified Müller‐Hinton II cation‐adjusted broth, enriched with 2% IsoVitaleX without chitosan or CSNPs, served as negative controls. After incubation, the tubes were observed for the turbidity as a visible sign of bacterial growth; no growth indicates the antibacterial properties of the reagent at that concentration. The assay was performed in triplicate, and the result repeated twice or more was considered.

#### 2.3.3 Minimum inhibitory concentration (MIC) assay

The turbidimetric method using different concentrations of chitosan and CSNPs was performed to assess the MIC value for each bacterial strain. Further double‐fold serial dilutions of CSNP (1 mg/ml) suspension were tested according to the methods described by Du et al. (2009) and Qi et al. (2004). In addition, serial concentrations of chitosan and CSNPs from 2 to 10 mg/ml were tested according to Divya et al. (2017). Briefly, under aseptic conditions, one test tube containing 10 ml CSNP (1 mg/ml) suspension in MH broth and a serial number of test tubes each containing 5‐mL sterile MH broth were specified for each strain. A series of double‐fold dilutions was prepared for CSNP suspensions via well mixing of 5 ml with the next tube containing 5 ml MH broth. Serial transformations and mixing through all the test tubes gave rise to serial double‐fold dilutions. 5 ml from the last dilution mixture was discarded to achieve the same volume in all tubes. Hence, each tube contained 5 ml test sample suspension with half of the previous concentration.

Nine more test tubes each containing 5 ml sterile MH broth were specified for each strain. For *F. noatunensis* subsp. *Orientalis*, the tubes contained modified Müller‐Hinton II cation‐adjusted broth, enriched with 2% IsoVitaleX. Serial concentrations from 2 to 10 mg/ml were prepared by suspending the accurately weighted chitosan or CSNP powders in its corresponding test tube (Divya et al., 2017). The bacterial suspensions were inoculated into the test tubes to achieve a final concentration of 1 × 10^5^ CFU/ml (Du et al., 2008). Similar concentrations of chitosan or CSNP suspensions served as the blank controls, and MH broth inoculated with each tested strain or modified Müller‐Hinton II cation‐adjusted broth, enriched with 2% IsoVitaleX inoculated with *F. noatunensis* subsp. *Orientalis,* served as negative controls. All the test tubes were incubated at the same conditions described above, and the lowest concentration of the reagent that inhibited the visible growth of each bacterium compared with its negative control was considered the related MIC value. The assay was performed in triplicate, and the result that repeated twice or more was considered (Du et al., 2009).

#### 2.3.4 Minimum bactericidal concentration (MBC) assay

The lowest concentration of CSNPs, which inhibits 99.9% of the bacterial growth on plates is considered the corresponding MBC value (Du et al., 2008; Qi et al., 2004). To assess the MBC values corresponding for each sensitive bacterial strains, samples of 100µl from bacteria/CSNP mixture showing no visible bacterial growth during MIC test were streaked on agar plates and incubated in a static incubator at the same conditions described above. The absence of the bacterial colonies on plates indicates the lack of living bacteria and the bactericidal effect at that concentration. On the other hand, growing of bacterial colonies on plates indicates the presence of living bacteria and the bacteriostatic effect at that concentration. The assay was performed in triplicate, and the results repeated twice or more were considered.

#### 2.3.5 UV‐vis absorption

To confirm the bacterial growth inhibition, the optical densities (OD) in the broth media of all the replicate test tubes corresponding to the sensitive isolates during MIC assay were measured using UV‐vis spectrophotometer at 600 nm (Ali, Rajendran, & Joshi, 2011). The lower absorbance capacity indicates less bacterial growth and vice versa. Blanks for the OD measurements were the same MIC blank controls. The antibacterial effect of each dose from CSNPs was expressed as the percentage inhibition (%) of the bacterial growth according to the following equation (Divya et al., 2017).

(1) $$\text{Growth}\text{Inhibition}P\text{ercent}(\mathrm{GI\%})=1‐\left(\frac{\text{OD}\text{sample}}{\text{OD}\text{control}}\right)\times 100$$

The growth inhibition per cent (GI%) of each replicate was calculated from the obtained OD_600_ measurements, and the results were expressed as mean percentages ± standard deviation (*SD*) in histograms comparing the dose‐ dependent and species‐specific antibacterial efficacy of the prepared CSNPs.

### 2.4 Assessment of the antifungal efficacy of CSNPs

The ability of CSNPs to inhibit the growth of the two fungal‐like oomycetes *Aphanomyces invadans* and *Saprolegnia parasitica* was investigated. *A. invadans* was isolated from clinically infected dwarf gourami (*Colisa lalia*), while *S. parasitica* was isolated from clinically infected rainbow trout (*Oncorhynchus mykiss*). They were identified molecularly by PCR and kept in our Microbank in the Clinical Division of Fish Medicine, University of Veterinary Medicine, Vienna, Austria. The identity of the *A. invadans* isolate was confirmed using Ainvad‐2F (5′‐TCATTGTGAGTGAAACGGTG‐3′) and Ainvad‐ITSR1 (5′‐GGCTAAGG TTTCAGTATGTAG‐3′) primers according to Vandersea et al. (2006). Sequences obtained from PCR products were subjected to BLAST search analysis against the GenBank database, which revealed 100% homology with the fragment of 18S ribosomal RNA, and internal transcribed spacer 1 (ITS 1) genes of *A. invadans*. For the molecular confirmation of *S. parasitica* isolate, ITS region sequencing was carried out by using universal primers ITS1 (5`‐TCCGTAGGTGAACCTGCGG‐3`) and ITS4 (5`‐TCCTCCGCTTATTGATATGC‐3`) as described by White, Bruns, Lee, and Taylor (1990). The resulted sequence revealed 100% homology with the *S. parasitica* ITS gene sequences in GenBank database.

*Aphanomyces invadans* was grown on glucose‐peptone (GP) agar after five days of incubation in a static incubator at 26°C (Shaalan et al., 2017). *S. parasitica* was grown on glucose–yeast (GY) agar after one to two days of incubation in a static incubator at 21 ± 1ºC (Yuasa, Kitancharoen, & Hatai, 1997). Stock cultures were kept in our laboratory on agar slopes immersed under mineral oil and were transferred and subcultured monthly on fresh media (Pottinger & Day, 1999).

The growth inhibitory effect of different concentrations of chitosan and CSNPs (1–10 mg/ml) was evaluated against the two oomycetes according to Shaalan et al. (2017) with some modifications. Briefly, two “24‐well microtitre” plates were specified for each oomycete, one used for chitosan and the other one used for CSNP suspensions. 1 ml of GP or GY broth was placed within a serial number of wells of each plate. Serial concentrations of chitosan or CSNP suspensions (1–10 mg/ml) were prepared by suspending the corresponding powder weight directly into its specific well prior to use. A small piece of 1 mm from the periphery of the growing mycelia of each oomycete was inoculated in each well containing its specific broth medium. Wells free from reagents served as controls, where the un‐inoculated wells served as blank controls and the inoculated wells served as negative controls. All the microtitre plates were incubated in a static incubator at the conditions specified for each oomycete as mentioned above. After incubation, the ocular density of each sample was compared with its negative control. The stronger antifungal concentration, which is the lowest concentration inhibited 90% of the visible mycelial growth in the broth, was recorded for each oomycete and considered the corresponding MIC_90_ (Ing et al., 2012). The assay was performed in triplicate, and the result that repeated twice or more was considered.

### 2.5 Ultrastructural time‐dependent CSNP/pathogen interaction

The interaction of CSNPs with the most sensitive bacterium, *Pseudomonas fluorescens*, and the fast‐growing fungal‐like oomycete, *S. parasitica*, was monitored by TEM to highlight the effect on their membranes compared with the untreated controls. Dynamics in the bacterial cell membrane morphology was observed after several incubation periods, while observation of the changes in *S. parasitica* mycelial membrane morphology was achieved after two days of incubation with CSNPs. Briefly, a single colony was inoculated in each one of five falcon tubes containing 5 ml Müller‐Hinton (MH) broth using a wire loop and incubated at 22ºC overnight in a shaker incubator (144 rpm) until the late exponential phase. Doubled MIC weights of CSNPs (Du et al., 2008) were accurately weighed and added directly into four of the test tubes containing growing bacteria. The fifth tube did not receive CSNPs and served as a control. All the tubes were re‐incubated at the same conditions described above for different incubation periods (30, 60, 120, 180 or >180 min). On the other hand, 1 ml GY broth was placed into two wells of a 24‐well microtitre plate, one well was supplied with doubled MIC weight of CSNPs, while the other well did not receive CSNPs and served as control. Small pieces of 1mm from the periphery of the growing mycelia were inoculated in each well, and then, the well plate was incubated at 21 ± 1ºC for two days. The fungal mycelia and the bacterial pellets were collected after each specific incubation period by centrifugation at 11, 600 × *g* for 15 min, resuspended in 1 ml phosphate buffer saline (PBS), centrifuged again and then processed for TEM.

All samples were fixed in 5% glutaraldehyde (in 0.1 M PBS) for 2–4 hr at 4°C. Post‐fixation was performed using 1% osmium tetroxide for 2 hr at 4°C followed by washing twice with PBS, gradual dehydration using alcohol series (70, 96 and 100%), soaking for 45 min in 1:1 mixture of glycidyl ether and propylene oxide, and then overnight incubation with 3:1 mixture of glycidyl ether and propylene oxide. The samples were embedded in a Spurr low‐viscosity embedding medium (i.e. gelatine capsules), and then, ultrathin sections were prepared with a diamond knife on an Ultracut Ultramicrotome. For contrast, the ultrathin sections were double‐stained on uncoated copper specimen grids using saturated uranyl acetate and lead citrate (Liu, Du, Wang, & Sun, 2004). The mounted grids were examined with EM 900 (Zeiss®, Oberkochen, Germany) and Image SP Viewer® software.

### 2.6 Cytotoxicity assay

#### 2.6.1 Fish cell lines and culture condition

The effect of the prepared CSNPs on the viability and survival of two epithelioid fish host cells was investigated. Chinook salmon (*Oncorhynchus tshawytscha*) embryo (CHSE‐269) cells and Epithelioma papulosum cyprini (EPC‐228) cells from carp (*Cyprinus carpio*) were used. They were cultivated in tissue culture plates containing minimal essential medium (MEM, Gibco) supplemented with 2% foetal bovine serum and were incubated at 20℃ for 24 hr until reaching a confluent monolayer. The approximate seeding density was 4 × 10^5^ and 1.5 × 10^5^ cells/cm^2^ for CHSE and EPC, respectively.

#### 2.6.2 Cytotoxicity and cell viability assessment

In vitro cytotoxicity of the prepared CSNPs was evaluated on the viability of both the selected fish cell lines to approve their safety for the living cells. The viability of the cells was estimated after 24 and 48 hr of incubation periods with several doses of the prepared CSNPs according to Qi, Xu, Li, Jiang, and Han (2005) with some modifications. Briefly, 100µl containing approximately 1.6 × 10^5^ CHSE cells or 6 × 10^4^ EPC cells was seeded into each well of 24‐well plate containing 1ml MEM and allowed to adhere at the same conditions described above. After reaching the confluent monolayer, all media were replaced with new media containing one of four different concentrations of CSNP suspension (1, 3, 5 or 7 mg/ml), except for the untreated negative control wells, and incubated for 24 hr at the same conditions mentioned above. Replicate plates were incubated for further 24 hr. The selected concentrations were chosen based on the observed MIC values.

Cytotoxicity of the selected CSNP doses was assessed by the trypan blue exclusion assay (Dodane, Amin Khan, & Merwin, 1999; Qi, Xu, Jiang, Li, & Wang, 2005). Briefly, after 24 hr and 48 hr of incubation periods, the culture media were removed and the cells were washed carefully by PBS to remove residual CSNPs. The cells were mixed 1:1 with 0.4% trypan blue dye (Sigma‐Aldrich, Austria) for one minute. For fixation, the stain was replaced with formalin 4% for 10 min. After fixation, the cells were rinsed three times with PBS and examined under an inverted microscope. The number of dead cells (stained blue) versus viable intact cells (unstained) was counted per one hundred cells (Menanteau‐Ledouble, Lawrence, & El‐Matbouli, 2018) in triplicate per each well (three fields). The per cent of surviving cells was calculated for each well, and the survival per cent (viability) was expressed as mean per cent ± *SD*. The assay was performed in triplicate with three replicate wells for each treatment and a control well per assay.

### 2.7 Statistical analysis

All data were expressed as means ± *SD* obtained from ≥3 independent experiments conducted on different days with at least one replicate per experiment. The obtained data were normally distributed and the statistical analysis was performed using SPSS 25 (IBM) software, for the covariance (ANOVA) (Dytham, 2011) with *post hoc* Tukey's test for the paired comparison of means. Statistical significance of differences from control values was taken at *p* < .05 evaluating the data at >95% confidence level. *p*‐values of <.01 and <.001 were considered highly significant and very highly significant, respectively.

## 3 RESULTS

### 3.1 Characterization of the prepared CSNPs

#### 3.1.1 Particle size and size distribution

**FIGURE 1:**
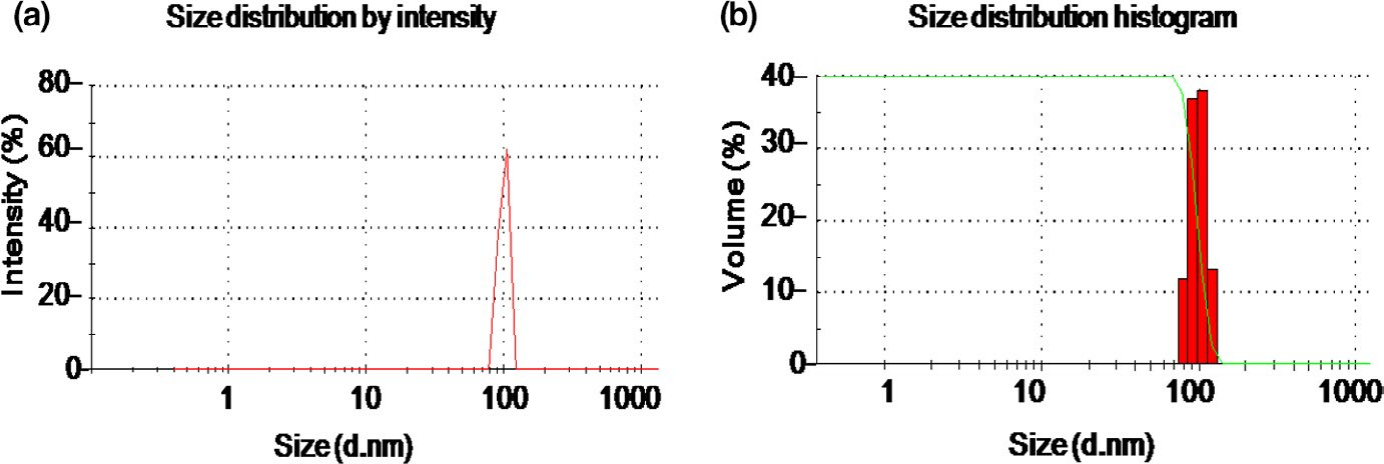
Characterization of the prepared CSNPs. (a) Particle size by intensity peak referring at 105 nm, and (b) particle size distribution including red column chart showing narrow size distribution range from 70 to 140 nm, and green disciplined Z‐shaped peak showing the homogenous distribution of the particles. All data were expressed as means ± *SD* (*n* = 3)

The mean size and size distribution profile of the prepared CSNPs are presented in Figure 1. The particles have a mean diameter of 105 nm (Figure 1a), with a narrow size distribution range from 70 to 140 nm and a polydispersity index of 0.6 (Figure 1b).

#### 3.1.2 Microscopic assay of CSNPs

**FIGURE 2:**
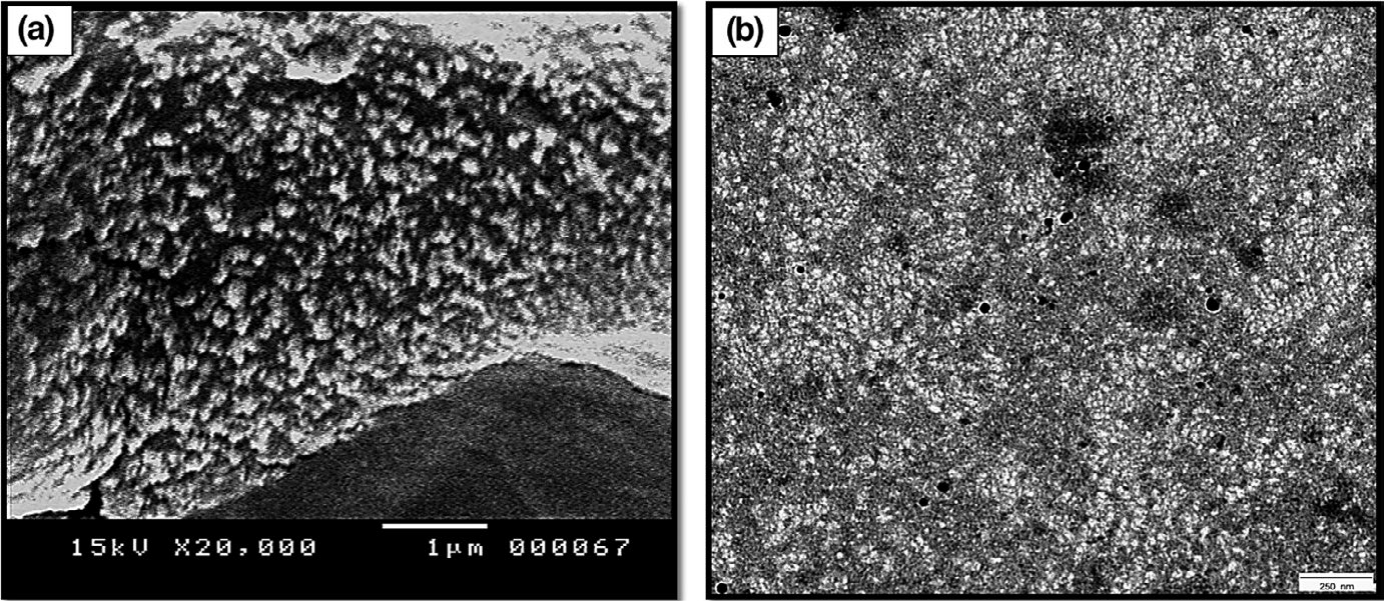
Microscopic assay of the synthesized CSNPs. (a) *SEM* micrograph (scale bar = 1 µm) showing homogenous spherical particles without agglomerations and (b) TEM micrograph showing homogenous spherical‐shaped particles (scale bar = 250 nm)

*SEM* micrographs scanned homogenous spherical particles without agglomerations (Figure 2a). TEM micrographs showed spherical particle shape with regular surfaces and homogenous sizes (Figure 2b). The scanned particles have a diameter range of 76.78–162.27 nm with an average of 107.65 nm.

### 3.2 Antibacterial effect of CSNPs.

#### 3.2.1 Bacterial growth inhibition

Both chitosan and CSNP powders did not affect the pH of the broth media; however, they explored different antibacterial activities. Chitosan suspension (1 mg/ml) could not inhibit any of the bacterial strains in the current study. On the other hand, CSNP suspension (1 mg/ml) inhibited the visible growth of six bacterial isolates including *P. fluorescens, Aeromonas hydrophila*, *Yersinia ruckeri, Pseudomonas putida, Aeromonas caviae* and *Aeromonas veronii* after overnight incubation. This dose, however, could not inhibit the growth of the other strains examined in the current study.

##### Minimum inhibitory concentration (MIC).

The turbidimetric method revealed that chitosan suspension was not effective as an antibacterial agent and could not inhibit the growth of any bacterial strain of this study. On the other hand, CSNP suspensions showed a considerable dose‐dependent, and species‐specific antibacterial effects against eleven of the tested sixteen strains, this is evident from the explored varied MIC values (Table 1). The lowest MIC concentration was recorded against *P. fluorescens* at 0.125 mg/ml, and the highest MIC value was recorded against *Pseudomonas aeruginosa* at 5 mg/ml. However, the further five tested isolates including Vibrios (*V. vulnificus, V. parahaemolyticus, V. harveyi, V. alginolyticus*) and *Citrobacter freundii* were highly resistant to CSNP suspensions, even 10 mg/ml could not inhibit their growth.

##### Minimum bactericidal concentration (MBC)

The lowest concentration of CSNPs, which was able to prevent 99.9% of the bacterial growth on agar plates, was recorded. CSNPs were bactericidal for seven strains and prevented their colonial growth. They were bacteriostatic for four further strains and could not prevent their colonial growth at distinct concentrations. The lowest MBC value was recorded against *P. fluorescens* at 1 mg/ml indicating the greatest sensitivity to CSNPs, while the highest MBC value was recorded at 7 mg/ml against *P. putida* indicating the highest resistance to CSNPs. However, the MBC values against *F. noatunensis* subsp. *orientalis, Edwardsiella ictaluri, A. caviae* and *A. veronii* were not assessed at all concentrations used during the current study (1–10 mg/ml) (Table 1).

##### The effect of CSNPs on bacterial growth

**FIGURE 3:**
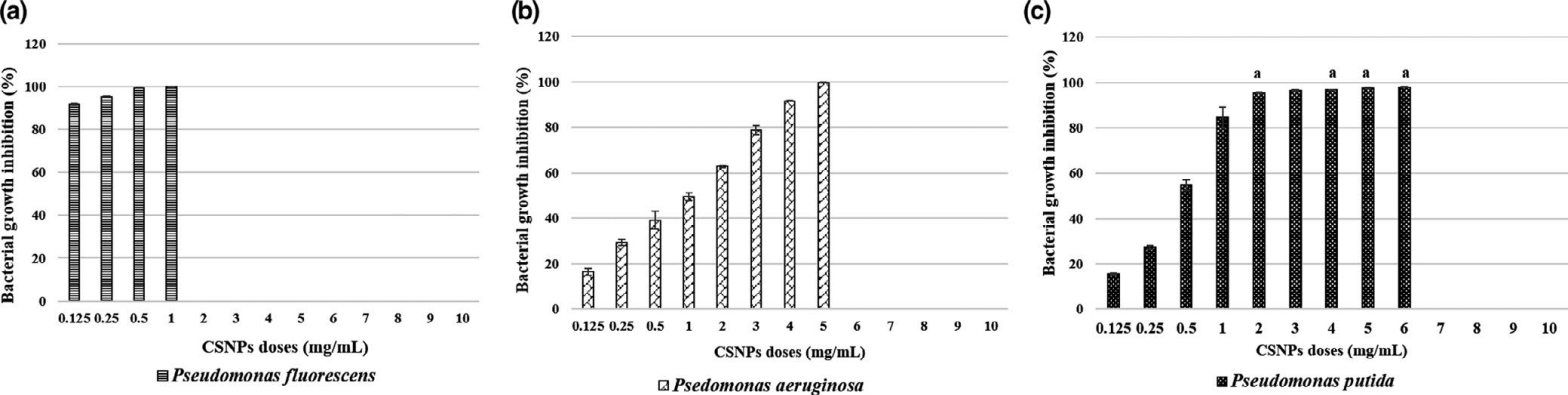
Per cent growth inhibition of *Pseudomonas* spp. reaching the MBC doses. (a) Per cent inhibition of *P. fluorescens*, (b) per cent growth inhibition of *P. auruginosa* and (c) per cent growth inhibition of *P. putida*. All data were expressed as means ± *SD* (*n* = 3), and similar letters indicate results are not significantly different

**FIGURE 4:**
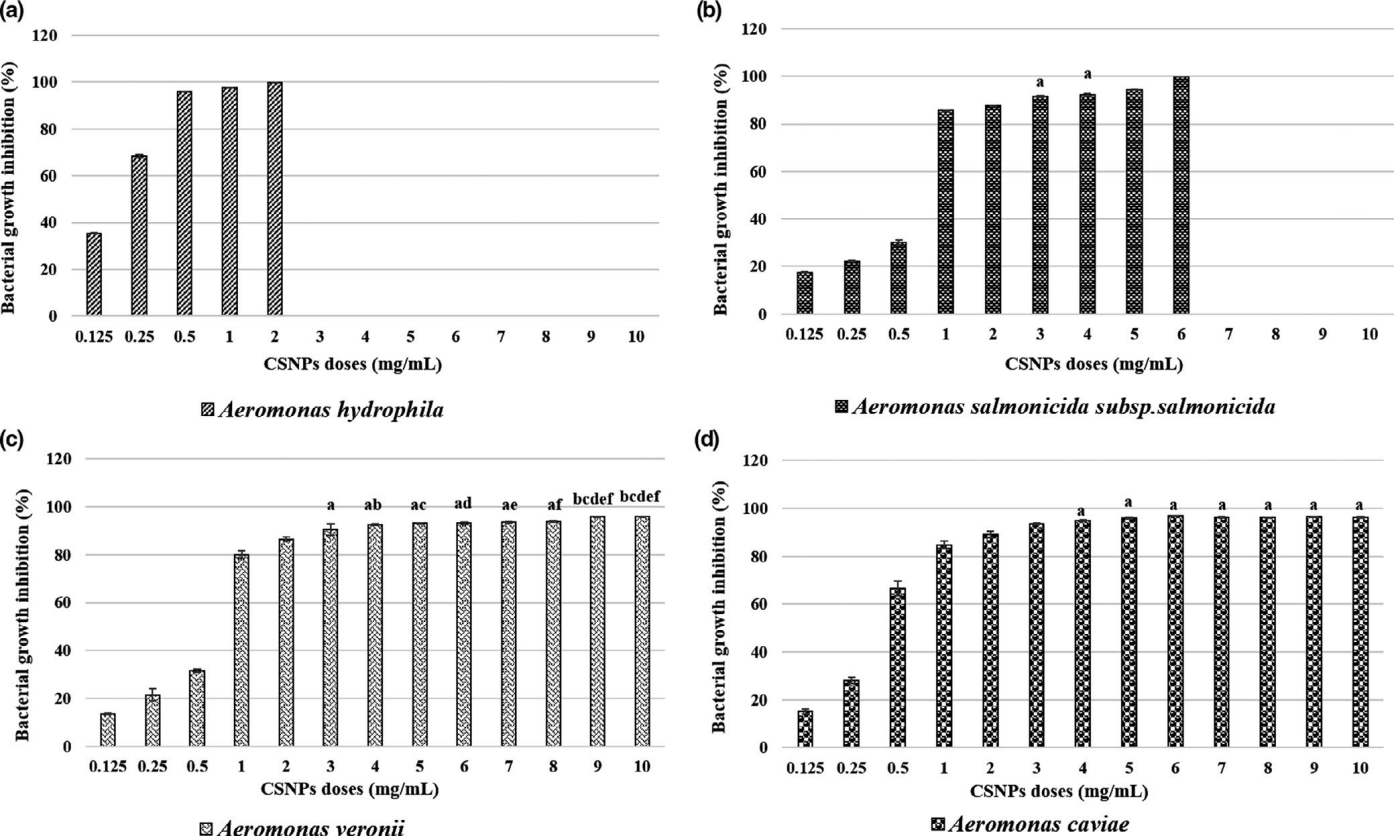
Per cent growth inhibition of *Aeromonas* spp. at all the examined CSNP concentrations or reaching the MBC dose. (a) Per cent growth inhibition of *A. hydrophila*, (b) per cent growth inhibition of *A. salmonicida* subsp. *salmonicida*, (c) per cent growth inhibition of *A. veronii* and (d) per cent growth inhibition of *A. caviae*. All data were expressed as means ± *SD* (*n* = 3), and similar letters indicate results are not significantly different

**FIGURE 5:**
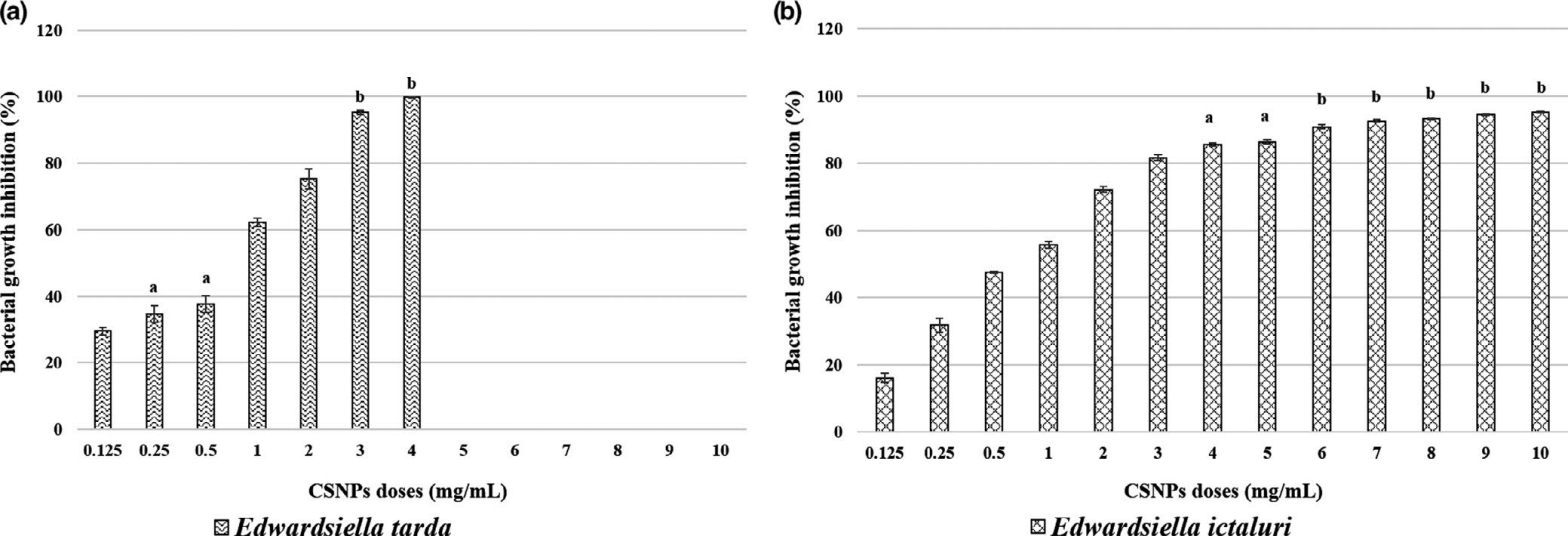
Per cent growth inhibition of *Edwardsiella* spp. at all the examined CSNP concentrations or reaching the MBC dose. (a) Per cent growth inhibition of *E. tarda*. (b) Per cent growth inhibition of *E. ictaluri*. All data were expressed as means ± *SD* (*n* = 3), and similar letters indicate results are not significantly different

**FIGURE 6:**
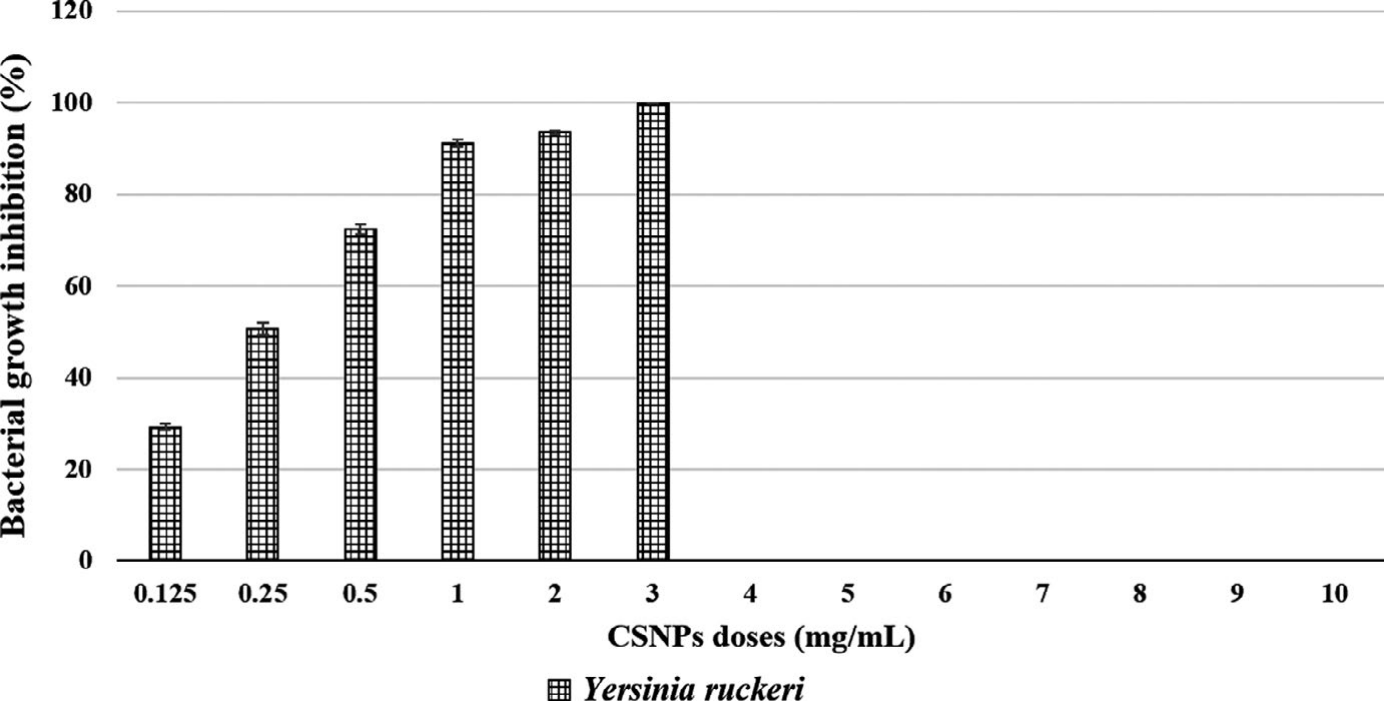
Per cent growth inhibition of *Yersinia ruckeri* at each examined CSNP concentration, reaching the MBC dose. All data were expressed as means ± *SD* (*n* = 3) (*p* < .002)

**FIGURE 7:**
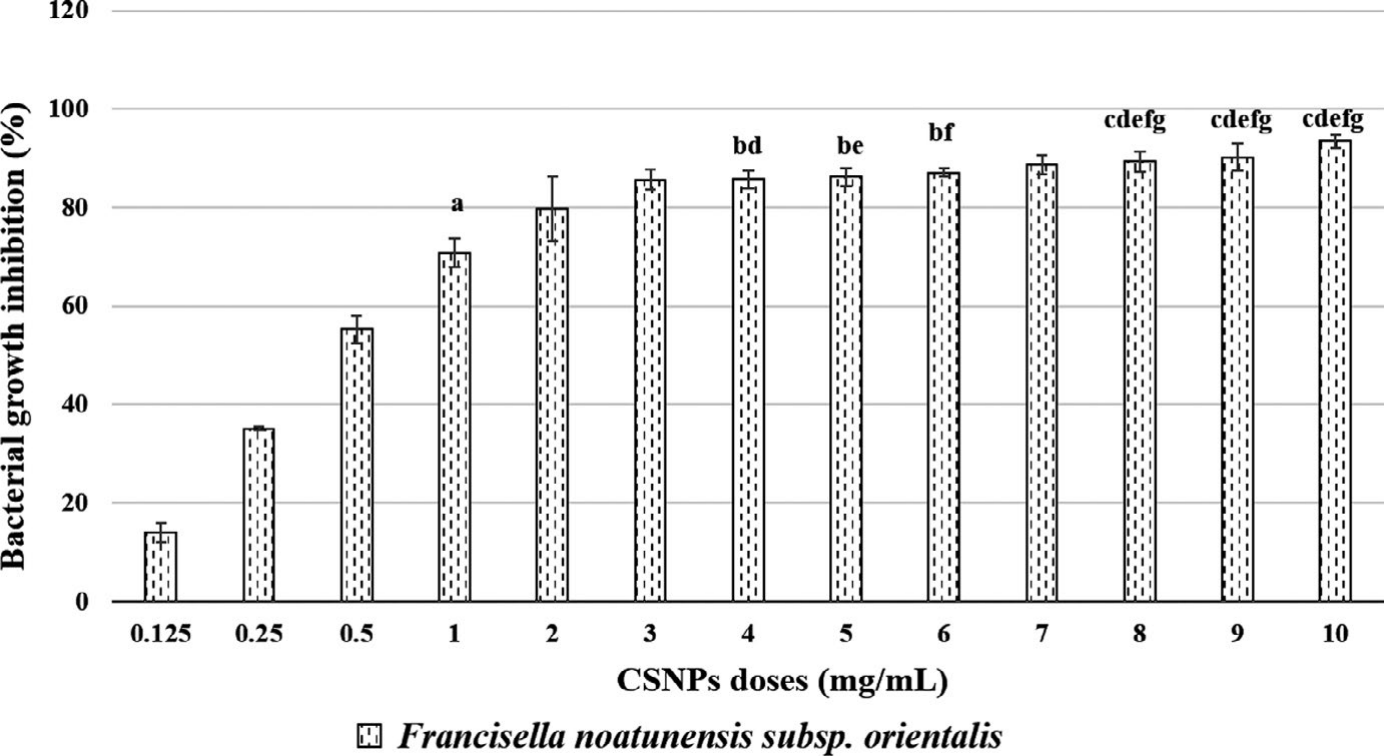
Per cent growth inhibition of *Francisella noatunensis* subsp. *orientalis* at all the examined CSNP doses. All data were expressed as means ± *SD* (*n* = 3), and similar letters indicate results are not significantly different

**FIGURE 8:**
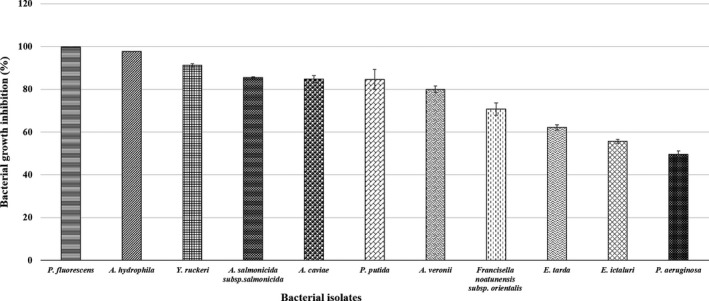
Descending arrangement of the bacterial sensitivity showing the growth inhibition ability of CSNPs at 1 mg/ml (*p* < .001)

The bacterial growth inhibition in the CSNP/bacteria mixtures was confirmed by their lower absorbance capacities at 600 nm after overnight incubation. For each bacterial strain, the mean per cent growth inhibition at all CSNP doses or reaching the corresponding MBC dose was assessed. The dose‐dependent, and the species‐specific antibacterial effects of the prepared CSNPs were evaluated showing their bactericidal or bacteriostatic effects (Figures 3, 4, 5, 6, 7). For all isolates, the growth inhibition per cent of the tested doses showed a high significant difference (*p* < .001) in comparison with that of the untreated controls. Additionally, the growth inhibition per cent of the sensitive bacterial isolates was displayed at a specific dose (1 mg/ml) showing the species‐specific‐dependent antibacterial effect of CSNPs (Figure 8).

### 3.3 Antifungal effect of CSNPs

No tested doses of chitosan suspension inhibited the growth of *A. invadans* or *S. parasitica*. On the other hand, CSNPs inhibited 90% of their growth in broth media. After incubation with serial concentrations of CSNPs (1–10 mg/ml), the mycelial growth was decreased gradually and the MIC_90_ values were recorded at 3 and 4 mg/ml for *A. invadans* and *S. parasitica*, respectively.

### 3.4 CSNP/pathogen interaction

**FIGURE 9:**
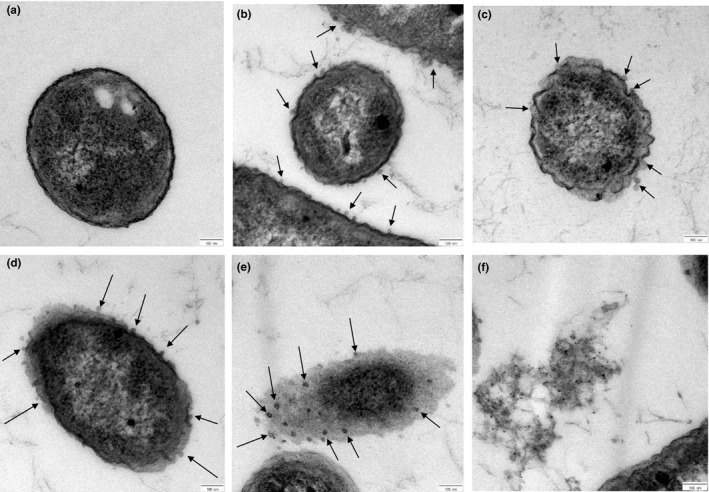
TEM photomicrographs (scale bar = 100 nm) of *P*. *fluorescens* cells after treatment with CSNPs (arrows) for different incubation periods. (a) Untreated cell, (b) cell treated for 30 min, (c) cell treated for 60 min, (d) cell treated for 120 min, (e) cell treated for 180 min and (f) cell debris after > 180 min

**FIGURE 10:**
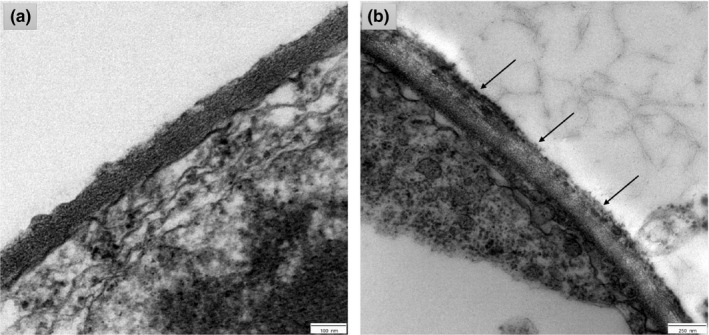
TEM photomicrographs showing the ultrastructure of *Saprolegnia parasitica* mycelial membrane after incubation with CSNPs. (a) Untreated membrane (scale bar = 100 nm). (b) Membrane treated for 48 hr (scale bar = 250 nm)

The time‐dependent interaction between CSNPs and *P. fluorescens,* and *S. parasitica*, revealed time‐dependent outer membrane alterations. TEM scanning showed the attachment of CSNPs to the outer membranes of *P. fluorescens* (arrows) after 30 min of incubation (Figure 9b). After 60 min of incubation, CSNPs induced degradation and rupture of the outer cell membranes; therefore, the cells start to shrinkage and appear in irregular shape compared with the control untreated cells (Figure 9a, c). After incubation for 2 hr, cell membranes were disrupted and leakage of the intracellular contents was observed (Figure 9d). Complete loss of the cell membranes was observed as a sign of complete fragmentation after 3 hr (Figure 9e). Cell debris was monitored after incubation for more than >3 hr indicating complete cell death (Figure 9f). Furthermore, TEM scanned the attachment of CSNPs on different sites of *S. parasitica* mycelial membrane (arrows) after 48 hr of incubation with CSNPs. In the attachment sites, the mycelial membrane was observed to be weak, disrupted, therefore gained light staining in comparison with the control untreated membrane (Figure 10a, b).

### 3.5 CSNP cytotoxicity assessment.

**FIGURE 11:**
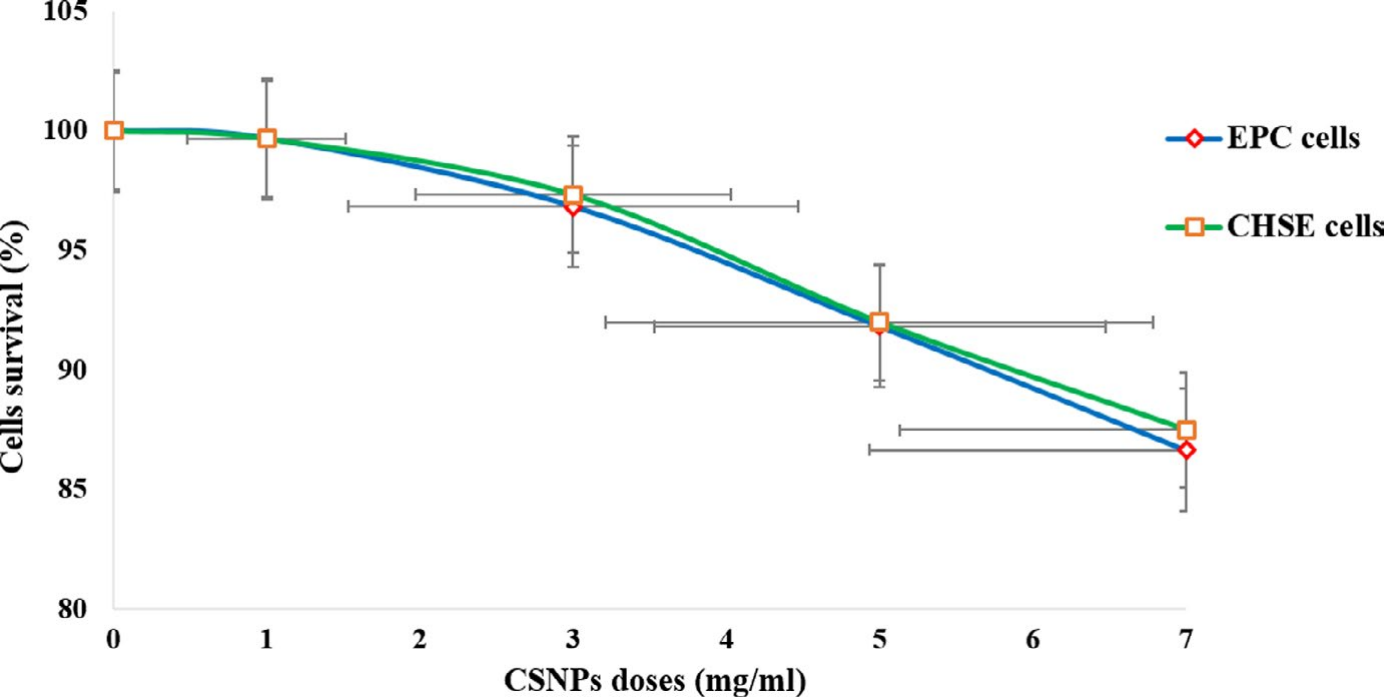
Cytotoxicity of CSNPs on EPC and CHSE cells assessed by the trypan blue exclusion assay. Cells viability is expressed as mean per cent ± *SD* (*n* = 3). No significant differences (*p* = .99) were observed on the viability of both cell lines at the treatments 1 mg/ml, or on the viability of CHSE cells at the treatment 3 mg/ml (*p* = .1). Significantly lower viability was observed on EPC cells at 3, 5 and 7 mg/ml (*p* < .003), and on CHSE cells at 5 and 7 mg/ml (*p* < .001)

The viability of CHSE and EPC cells was variable in relation to the assessed CSNP doses indicating dose‐dependent cytotoxicity. For both cell types, no significant differences (*p* = .99) were observed on the viability of the treatment 1 mg/ml compared with the non‐treated negative control cells. On the other hand, a dose‐dependent reduction in cell viability was observed after 24 hr of incubation with the higher CSNP concentrations (3, 5 and 7 mg/ml). EPC cells were more sensitive and explored high significant (*p* < .003) dose‐dependent lower viability at 3, 5 and 7 mg/ml. CHSE cells were more resistant with significant lower viability (*p* = .1) observed at the 3 mg/ml dose, while a very high significantly lower viability (*p* < .001) was recorded at the higher doses (5 and 7 mg/ml) in comparison with the untreated cells. The viability of the cells at all treatments was higher than 86% and 87% for EPC and CHSE, respectively. No obvious change was observed in the morphology of the treated cells compared with the control cells. Cell survival was expressed as mean per cent ± *SD* and represented in a line chart (Figure 11). No additional toxicity was observed on both cell lines upon prolongation of the incubation period up to 48 hr (*p* > .7).

## 4 DISCUSSION

In the current study, the ionic gelation method was followed to obtain chitosan nanosuspension, as it is a safe, controllable and fast method that depends on the ionic interaction between the positively charged amino groups of the acidic chitosan solution and the negatively charged polyanions of the aqueous STPP solution. The continuous magnetic stirring at room temperature allows the intermolecular cross‐linking between the oppositely charged molecules. Chitosan, which is soluble only in acidic media, was not adequately dispersed and was precipitated down when added to the basic broth media. Hence, chitosan could not interact with the microbial cells or inhibit their growth. This is in an agreement with Du et al. (2008), Du et al. (2009) who reported no antibacterial effect of chitosan suspensions in the basic MH broth. The bulk chitosan macromolecules have poor cellular uptake and remain extracellularly, which limits their antimicrobial effects (Ma & Lim, 2003). However, according to other studies, the antimicrobial effect of chitosan in acidic media is based mainly on the ability of the released polycations to attach with the negatively charged cell membrane components of many microorganisms disrupting their functions and causing cell death (Qi et al., 2004; Raafat, Bargen, Haas, & Sahl, 2008). Similarly, Cheng and Li (2000) reported that powdered chitin, chitosan or even whole crab shells were not effective as antimicrobial agents in all tests, while chitosan solution in acetic acid exhibited antimicrobial effects against some bacterial and fungal pathogens. Based on previously reported studies, the antimicrobial tests in our study were conducted using suspensions without any acidic solvents (e.g. acetic acid) to avoid their interference with the antimicrobial effect of chitosan or CSNPs, as the organic acids were reported to have different degrees of antibacterial abilities (El‐Shenawy & Marth, 1992). Additionally, acetic acid is well known by its antimicrobial properties (Breidt, Hazes, & Mcfeeters, 2004; No, Park, Lee, & Meyers, 2002; Roe, O’Byrne, McLaggan, & Booth, 2002) and is commonly used as an antiseptic agent in medicine (Ryssel et al., 2009). In earlier studies, the growth of *Pseudomonas* spp. was inhibited by the acidic chitosan (Balicka‐Ramisz, Wojtasz‐Pajak, Pilarczyk, Ramisz, & Laurans, 2005; Chung et al., 2004; Devlieghere, Vermeulen, & Debevere, 2004; Younes, Sellimi, Rinaudo, Jellouli, & Nasri, 2014), chitosan oligomers (No et al., 2002), chitosan/glucose complex (Kanatt, Chander, & Sharma, 2008b), N‐acetyl chitooligosaccharides (Benhabiles et al., 2012) and chitosan/mint extract mixture (Kanatt, Chander, & Sharma, 2008a). Similarly, the growth of *A. hydrophila* and *E. ictaluri* was inhibited with chitosan and chitosan oligosaccharide lactate solutions (Yildirim‐Aksoy & Beck, 2017).

Our results revealed that CSNPs (105 nm) exhibited an active dose‐dependent and species‐specific antimicrobial efficacy. However, the obtained data may differ from some other previously reported studies and this is likely due to the differences in materials, isolates, CSNP size and the experimental conditions, including CSNP preparation method, and the way of their applying against pathogens. Regarding their antibacterial properties, CSNPs explored bactericidal effect against seven bacterial strains, and bacteriostatic effect against four further strains examined during the current study, and the OD_600_ measurements reaffirmed the reduction in the bacterial growth of these sensitive strains after overnight incubation. CSNPs showed bactericidal effect against all the *Pseudomonas* spp. examined in the current study, and *P. fluorescens* was the most sensitive. Similarly, Divya et al. (2017) reported that CSNPs (120 nm) inhibited the growth of 95% of *P. aeruginosa* at a high dose of 10 mg/ml. Conversely, Abdel‐Razek (2019) reported that *P. aeruginosa* was more sensitive than *P. fluorescens* to CSNPs (35nm). Against *Aeromonas* spp., the prepared CSNPs exhibited bactericidal effects against both of *A. hydrophila* and *A. salmonicida* subsp. *salmonicida*, while elicited bacteriostatic effect against *A. veronii* and *A. caviae*. The most sensitive *Aeromonas* spp species was *A. hydrophila*, while both of *A. veronii* and *A. caviae* showed the highest resistance to CSNPs. This is in disagreement with Abdel‐Razek (2019), who reported bactericidal effect of CSNPs (35 nm) against *A. veronii*. The variation of the observed effect against *A. veronii* in both studies could be due to using different sizes of CSNPs.

In addition, CSNPs were bactericidal against *E. tarda* and *Y. ruckeri,* while they were bacteriostatic against *E. ictaluri* and *F. noatunensis* subsp. *orientalis*. However, they did not exhibit any antibacterial effects against all the four selected *vibrio* isolates, or against *C. freundii,* as they could not inhibit their growth at all concentrations tested during our study. On the contrary, the growth of *Vibrio* spp. was inhibited by chitosan (Benhabiles et al., 2012), chitosan oligomers (No et al., 2002) and chitosan microparticles (Jeon, Oh, Yeo, Galvao, & Jeong, 2014). Chitosan cross‐linked with STPP beads entrapped with *P. putida* for phenol degradation (Hsieh, Huang, Lin, Chen, & Lin, 2008).

Unlike bacteria, fungi are less sensitive to chitosan, and the difference between fungal strains that are sensitive or resistant to chitosan is less clear and less evidenced (Verlee, Mincke, & Stevens, 2017). In agreement with previous studies, the prepared CSNPs (105 nm) exhibited a fungistatic effect against both of *A. invadans* and *S. parasitica* at high MIC_90_ values. Earlier, chitosan and CSNPs exhibited antifungal effects against several fungal strains, including *Trichophyton mentagrophytes*, *Microsporum canis, Fusarium solani* and *Candida* spp. (Balicka‐Ramisz et al., 2005; Ing et al., 2012; Tayel et al., 2010). CSNPs (35 nm) inhibited the growth of *Aspergillus flavus*, *Mucor sp*., *Candida sp*., *A. niger*, *A. fumigatus* and *Fusarium sp*. (Abdel‐Razek, 2019). It is worth mentioning that the antifungal effect of chitosan is ATP‐dependent and is highly affecting with the type of the fungus owing to the differences in the fluidity of the fungal cell membranes (Palma‐Guerrero et al., 2010). Additionally, the membranes of chitosan‐sensitive fungi possess unsaturated fatty acids (the main tool for fungal classification) more than the chitosan‐resistant strains (Verlee et al., 2017). Moreover, fungi possess chitin or chitosan in their cell wall components, such as *Aspergillus niger*, are more resistant to chitosan (Allan & Hadwiger, 1979). Therefore, chitosan exhibits species‐specific antifungal effect against specific families.

Corresponding to the TEM photomicrographs of CSNP/pathogen interaction obtained from our study, the polycationic CSNPs, with their small particle size and high positive surface charges, were able to absorb tightly and interact electrostatically with the negatively charged pathogenic plasma membranes, which interfere with their integrity and functions. The prepared CSNPs attached to the bacterial cell membranes within 30 min, and the cell death was confirmed after > 3 hr of incubation period. Similar time‐dependent disruptions were monitored on *S. choleraesuis* and *E. coli* K88 via atomic force microscopy after similar incubation periods (Du et al., 2009; Qi et al., 2004), and on *A. hydrophila* via TEM microscopy after 24 hr of incubation with CSNPs (Abdel‐Razek, 2019). The bacterial cell death occurs via the alteration of the cell membrane permeability, which inhibits the transportation of the nutrients and causes leakage of the intracellular components (Qi et al., 2004). In addition, the CSNPs attached to numerous sites at the *S. parasitica* hyphal membrane after 48 hr of incubation period, affecting its normal growth and development. This is in agreement with Muzzarelli et al. (2001) who displayed *SEM* and TEM micrographs showing frayed, expanded hyphal membranes of *S. parasitica* with morphologically altered internal organelles upon coating with chitosan. Similarly, Tayel et al. (2010) displayed *SEM* micrographs showing swelled, asymmetric and rough hyphae of *C. albicans* with wall lyses after prolonged exposure to chitosan. This study is the first report on the ultrastructural changes in *S. parasitica* hyphal membrane after the exposure to CSNPs.

Cytotoxicity of the prepared CSNPs was evaluated on CHSE and EPC cell lines by following trypan blue exclusion assay, which is a selective staining technique comparing the viability of the treated cells with that of the untreated control cells. No significant cytotoxicity was observed on CSNPs (1 mg/ml) (*p* > .99), while poor dose‐dependent cytotoxicity was elicited by the higher concentrations (3, 5 and 7 mg/ml), which is the concentration range needed to induce a bactericidal effect in several of the bacterial isolates tested. CSNPs up to 7 mg/ml were poorly cytotoxic and cause less than 14% cell death (>86% viability) with no obvious changes in the morphology of the treated cells compared with the control cells. This is in an agreement with some previous studies that reported low toxicity of chitosan and CSNP complexes on fish cell lines at high doses. The viability of sea bass kidney cell line was more than 90% without significant change in their morphology after the incubation with CSNP/pDNA complexes (Rajesh Kumar et al., 2008; Rajeshkumar et al., 2009; Vimal et al., 2014) even at high concentrations up to 5 mg/ml (Vimal et al., 2012), which approves their safety at higher doses. On human cell lines, CSNPs (25‐100µg/ml) exhibited low toxicity on the normal hepatic cells (Qi, Xu, Jiang, et al., 2005), CSNPs (0.25–1 mg/ml) played a limited role in the apoptosis of the hepatic cancer cells (Liang et al., 2011), whereas CSNPs (< 0.741 mg/ml) did not affect the viability of lung carcinoma cells (Huang, Khor, & Lim, 2004). A 12 hr of incubation period with CSNPs (1‐5µg/ml) enhanced the growth and proliferation of both the mammalian normal and tumour hepatic cells in a dose‐dependent manner (Yang et al., 2014). It is worth mentioning that the positively charged CSNPs exhibited specific and high toxicity against tumour cancer cells having more negative charges on their membranes, while they exhibited low toxicity against normal cells (Huang et al., 2004; Qi, Xu, Jiang, et al., 2005; Qi, Xu, Li, et al., 2005; Rejinold et al., 2011).

No significant (*p* > .7) additional cytotoxic effect was observed upon prolonged exposure time (from 24 to 48 hr). This is in agreement with Loh, Saunders, and Lim (2012) who reported no increase in the cytotoxicity of CSNPs, particularly at low concentrations (< 0.05% w/v), on the human intestinal cells upon extension of the exposure time (up to 72 hr). On the contrary, Qi, Xu, Li, et al. (2005) reported significantly higher cytotoxicity of CSNPs (25–100 µg/ml) against human gastric carcinoma cells upon extending the exposure time from 24 to 48 hr. Prolongation of the exposure time from 4 to 24 hr revealed higher toxicity of CSNPs (0.5% w/v) against the human liver cells (Loh, Yeoh, Saunders, & Lim, 2010), while the lower concentration (0.025% w/v) promoted the recovery of the human intestinal cell viability (Loh et al., 2012). This is as referring to the different sensitivities between the different cell types towards CSNPs, and it is also the consent of the safety of CSNPs to the normal cells.

## 5 CONCLUSIONS

CSNP (105 nm) suspensions demonstrated in vitro dose‐dependent and species‐specific antimicrobial efficacy against the most common bacterial and oomycete fish pathogens. They exhibited potential antibacterial efficacy against *P. fluorescens*, *A. hydrophila* and *Y. ruckeri*. Additionally, they could combat *P. aeruginosa, P. putida, E. tarda* and *A. salmonicida* subsp. *salmonicida* using higher doses. Moreover, CSNPs could interact against and weaken the viability of *E. ictaluri, F. noatunensis* subsp. *orientalis, A. caviae, A. veronii, A. invadans* and *S. parasitica*. No significant cytotoxicity was observed on CSNPs (1 mg/ml), while a low dose‐dependent cytotoxicity was observed at higher doses (3, 5 and 7 mg/ml). Hence, it is anticipated that CSNPs could be incorporated for the disease management in aquaculture. However, further in vivo studies are still required to investigate the antimicrobial efficacy of CSNPs on the living fish.

## 6 CONFLICT OF INTEREST STATEMENT

The authors declare that they do not have any commercial associations, current and within the past five years, that might pose a potential, perceived or real conflict of interest.

## Data Availability

All data generated or analysed during this study are included in this published article.
